# Optical characteristics of type-II hexagonal-shaped GaSb quantum dots on GaAs synthesized using nanowire self-growth mechanism from Ga metal droplet

**DOI:** 10.1038/s41598-021-87321-9

**Published:** 2021-04-08

**Authors:** Min Baik, Ji-hoon Kyhm, Hang-Kyu Kang, Kwang-Sik Jeong, Jong Su Kim, Mann-Ho Cho, Jin Dong Song

**Affiliations:** 1 Department of Physics, Yonsei University, Seoul, 03722 Korea; 2 Center of Opto-Electronic Convergence Systems, Korea Institute of Science and Technology, Seoul, 02792 Korea; 3 Quantum-Functional Semiconductor Research Center, Dongguk University, Seoul, 04620 Korea; 4 Department of Physics, Yeungnam University, Gyeongsan, 38541 Korea

**Keywords:** Molecular self-assembly, Quantum dots, Synthesis and processing, Nanowires

## Abstract

We report the growth mechanism and optical characteristics of type-II band-aligned GaSb quantum dots (QDs) grown on GaAs using a droplet epitaxy-driven nanowire formation mechanism with molecular beam epitaxy. Using transmission electron microscopy and scanning electron microscopy images, we confirmed that the QDs, which comprised zinc-blende crystal structures with hexagonal shapes, were successfully grown through the formation of a nanowire from a Ga droplet, with reduced strain between GaAs and GaSb. Photoluminescence (PL) peaks of GaSb capped by a GaAs layer were observed at 1.11 eV, 1.26 eV, and 1.47 eV, assigned to the QDs, a wetting-like layer (WLL), and bulk GaAs, respectively, at the measurement temperature of 14 K and excitation laser power of 30 mW. The integrated PL intensity of the QDs was significantly stronger than that of the WLL, which indicated well-grown GaSb QDs on GaAs and the generation of an interlayer exciton, as shown in the power- and temperature-dependent PL spectra, respectively. In addition, time-resolved PL data showed that the GaSb QD and GaAs layers formed a self-aligned type-II band alignment; the temperature-dependent PL data exhibited a high equivalent internal quantum efficiency of 15 ± 0.2%.

## Introduction

Group III–V materials with direct bandgaps have been actively studied as novel photonic materials because their electrical and optical characteristics can be controlled by changing their structure and dimensions^1–4^. In particular, many researchers have studied Sb-based III–V materials for a wide range of applications in the next generation of technology in a telecommunication society^1^. Furthermore, the charge separation in these materials is significantly dependent on the type of band alignment because differences in band alignment case changes in several physical parameters, such as the recombination rate and emission wavelength. Owing to this clear dependence, the optically modulated characteristics resulting from the band alignment are continuously researched in three-dimensional bulk systems, as well as on one-dimensional nanowires or zero-dimensional quantum dots (QDs). Considering the dimensions and sizes of the materials, a QD is an excellent system for analyzing zero-dimensional quantum effects.

In this study, we synthesized a GaSb QD on a GaAs substrate using a self-grown method by droplet epitaxy^5^ with a molecular beam epitaxy (MBE) system^2, 3^. We investigated the optical properties of the QDs, which are dependent on the type-II band alignment of the GaSb/GaAs heterostructure; this structure differs from the type-I band structure of InAs/GaAs^6–12^. Charge separation can occur more easily in GaSb QDs because GaSb QDs form a type-II band alignment with GaAs. The structure of the GaSb QD shows that it has a zinc-blende crystal structure with a hexagonal shape. We characterized well-grown GaSb QDs using power-dependent and temperature-dependent photoluminescence (PL) measurements. The peak shifts to higher energies clearly suggested type-II band alignment. Furthermore, we cross-verified that the GaSb QD and GaAs layers formed a type-II band alignment using time-resolved PL (TRPL) data. Considering the superior PL characteristics resulting from the type-II band alignment, the equivalent internal quantum efficiency (E-IQE) of the GaSb QDs was ~ 15% when the intensity ratio is between 300 and 14 K. In conclusion, GaSb QDs were successfully grown through the nanowire self-growing mechanism on GaAs with a type-II band alignment, and a high equivalent internal quantum efficiency (IQE) was confirmed using temperature-dependent PL data.

## Result and discussions

**Figure 1:**
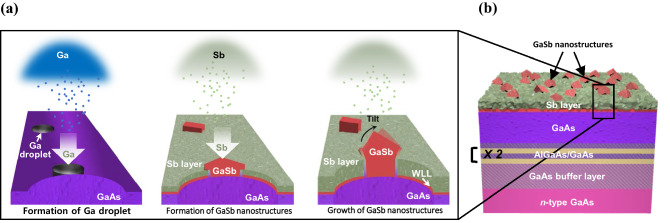
Schematic of (**a**) self-growth mechanism of the GaSb QD using Ga droplet without GaAs capping layer, and (**b**) GaSb QD structure on GaAs wafer.

GaSb QDs were grown on n-type GaAs (001) wafers using an MBE system. First, to reduce the native defects on the GaAs substrate, we sequentially grew a 200-nm-thick GaAs buffer, a short-period superlattice (SPS) structure with two alternate layers (5 nm-thick Al_0.3_Ga_0.7_As and 5-nm thick GaAs), and a 75-nm-thick GaAs layer at a growth temperature of 580 °C^13–15^. Then, we cooled the substrate to 350 °C with the valve of the As cracker cell and the main shutter (MS) open to prevent the vaporization of the As buffer layer. Afterward, we closed the valve of the As cracker cell and the MS because the GaAs substrate was thermally stable at temperatures of < 350 °C, as shown in Supporting Figure S1^16–18^. Next, a Ga droplet was formed on top of the GaAs layer in an ultra-high vacuum of less than 1 × 10^−9^ Torr at 250 °C for 10.6 s, as shown in Supporting Figure S2. The time was equivalent to the growth time of five-monolayer (ML) GaAs. After cooling the substrate to 180 °C, the Sb dimer was supplied to the Ga droplets at 180 °C for 5 min, resulting in the diffusion of Sb to the Ga droplets, as shown in Fig. 1a. Thus, the GaSb QDs were grown by supplying Sb to the Ga droplets on the GaAs surface, as shown in Fig. 1b. For the QDs without a capping layer, the growth process was complete. A two-step process was used to prevent the QD from melting while the GaAs capping layer was grown. First, GaAs was grown on top of the surface at 280 °C to prevent the GaSb QDs from melting without stopping the growth of the capping layer. Next, the substrate temperature was increased to 500 °C for the growth of an additional GaAs capping layer under the optimized conditions. As a result, a ~ 17-nm-thick GaAs capping layer was grown. The main parameters of the growth conditions, consisting of the substrate temperature (T_s_), beam equivalent flux (FAs), and growth time (t_g_), were optimized, as shown in Supporting Figure S1.

**Figure 2:**
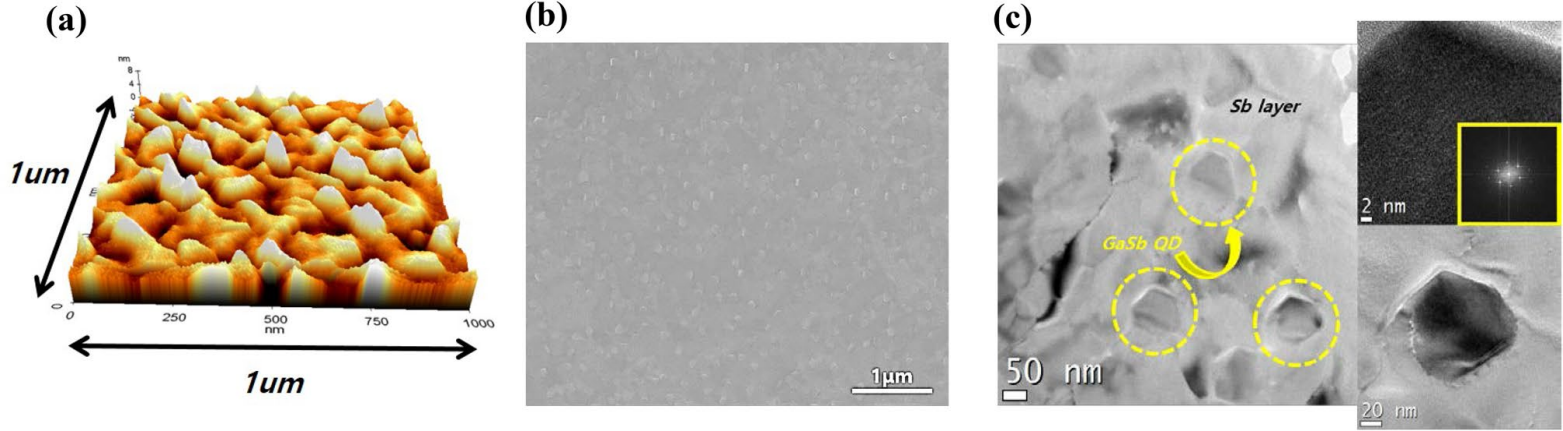
(**a**) AFM, (**b**) top-view SEM, and (**c**) TEM images of self-grown GaSb QD sample without GaAs capping layer to confirm the density and shape of GaSb QD.

To confirm the formation of QDs, we measured the structural and morphological characteristics of the sample without the GaAs capping layer using atomic force microscopy (AFM), scanning electron microscopy (SEM), and transmission electron microscopy (TEM). First, the surface morphology of the samples was analyzed using AFM to confirm the shape of the QDs. The AFM data in Fig. 2a clearly showed the morphological shape of the film surface containing the QD and the Sb layer, with a root mean square roughness (R_q_) of ~ 3 nm. Second, SEM and TEM were used to confirm the shape, distribution density, and diameter of the QDs. From the SEM and TEM data shown in Fig. 2b,c, an average diameter of 100 ± 5 nm was obtained for the QD. Focusing on the zone axis in the [111] direction, it was found that the nanostructure of the GaSb QD was neither spherical nor pyramidal; rather, the QD had a nanowire-like pillar shape with a zinc-blende structure. The density of the QDs was less than 10 μm^−2^, as shown in Fig. 2b,c. Furthermore, considering Fig. 2c and Supporting Figure S2, the GaSb nanostructure was formed via the self-catalyzed nanowire formation mechanism^5^, rather than the Stranski–Krastanov growth mechanism^17–20^. Specifically, the Ga droplet was formed in 10.6 s, equal to the deposition time of the 5-ML GaAs, as shown in Supporting Figure S2. Subsequently, a 40-nm-thick Sb layer was deposited on the GaAs surface, confirmed by the cross-sectional energy-dispersive X-ray (EDX) analysis of the outside of the QDs, as shown in Supporting Figure S3, where GaSb nanowires were grown under the growth conditions of the diffusion-limited process for the Sb atom. Because the migration of Sb was slow at 180 °C^21^, the amount of Sb that diffused into the Ga droplet was insufficient. In this case, the Ga droplet was used as a self-catalyst for QD growth, similar to nanowire growth. During the growth of the QDs, the wetting-like layer (WLL) observed around the GaSb nanostructure was not attributed to the growth of the GaSb QDs. Most of the Sb is considered to have vaporized during the increase in the substrate temperature up to 280 °C, and the origin of the WLL was attributed to the intermixing of residual Sb around the GaAs layer (see the inset of Figure S3).

**Figure 3:**
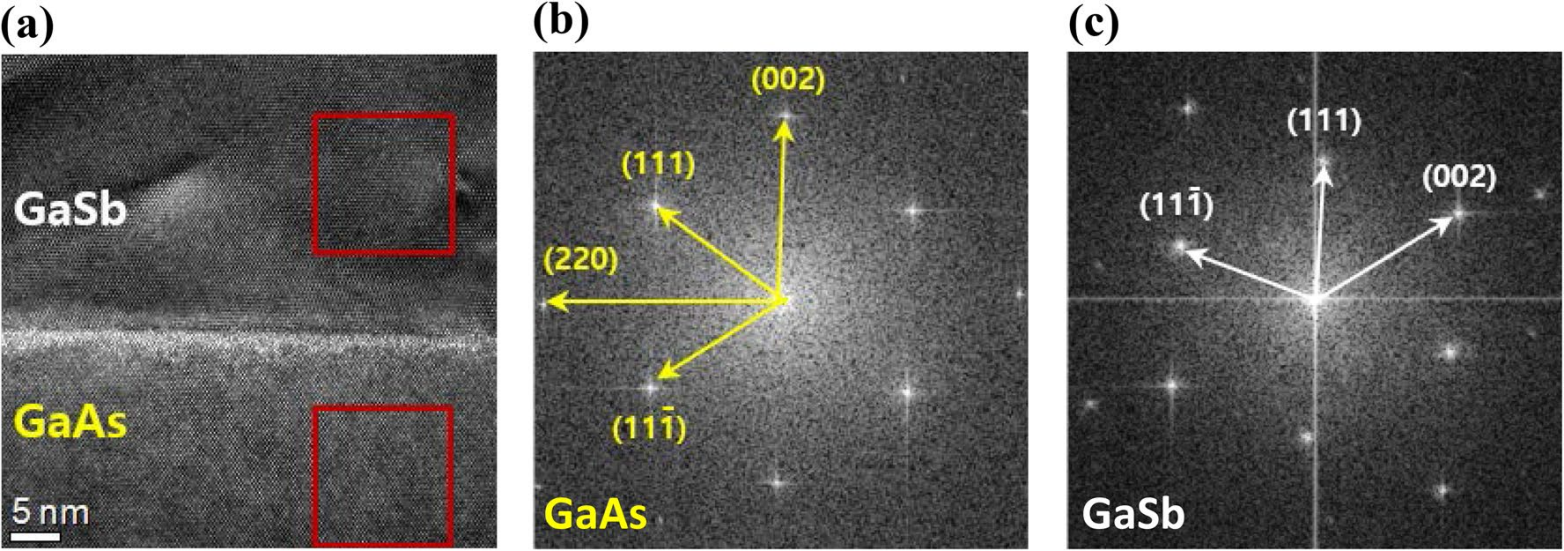
(**a**) Cross-sectional TEM images of the GaSb QD and GaAs layer, and selected-area diffraction patterns of (**b**) GaAs layer and (**c**) GaSb QD for confirming change of growth direction in GaSb QD.

To further investigate the nanostructure of the samples, we analyzed the cross-sectional TEM images, as shown in Fig. 3a, where a clear difference was observed. The top and bottom areas of the image indicate the GaSb QD and GaAs layer, respectively. Each red square from the bottom to the top shown in Fig. 3a was processed by fast Fourier transform (FFT), as shown in Fig. 3b,c. Based on the FFT images in Fig. 3b,c, the elongated direction of the QDs was not parallel to the [001] direction of the GaAs substrate. The cross-sectional image revealed that the QDs grown along the [111] direction of the GaAs layer had a zinc-blende structure. Using the reciprocal lattice constants of the GaAs layer in each direction, which were 2.93 nm^−1^, 2.96 nm^−1^, and 3.41 nm^−1^ from the selected-area diffraction (SAD) pattern, the diffracted plane was indexed based on the reported data, as shown in Fig. 3b^22–24^. Moreover, using the reciprocal lattice constants of GaSb in each direction, which were 2.60 nm^−1^, 2.71 nm^−1^, and 3.17 nm^−1^, the diffracted plane was indexed, as shown in Fig. 3c. Based on the reported lattice constants of (111), (11-1), and (002), we indexed the plane of the reciprocal lattice following the length of each direction, as shown in Fig. 3c. By comparison with the reported lattice constant of GaSb^25, 26^, we confirmed that the difference in the lattice constant of GaSb QDs was ~ 1.8%. From this result, we suggest that owing to the high lattice mismatch between GaAs and GaSb (> 7%), the growth direction of the GaSb QDs was changed to reduce the strain, allowing the most stable growth of the QDs. In this process, the strain was reduced by approximately 1.8% along the [111] direction. As a result, we can confirm that the QD is well grown along the [111] direction of GaAs with ~ 1.8% lattice strain. The GaSb QDs were thus successfully grown by a nanowire formation mechanism with a limited amount of group III material.

**Figure 4:**
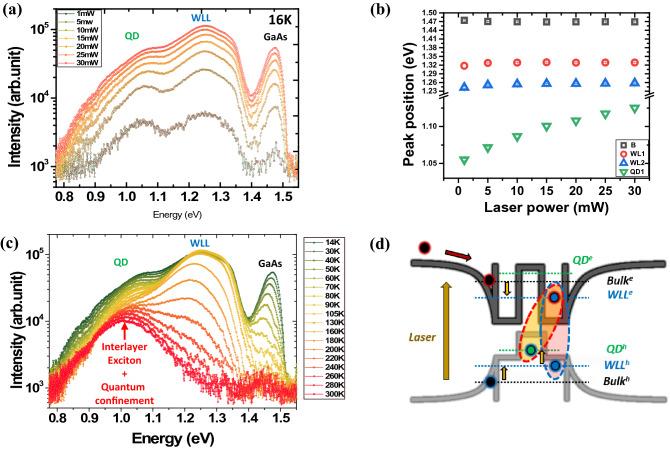
(**a**) Power-dependent PL spectra at 16 K, (**b**) peak energy shift data from the laser power of 1–30 mW, (**c**) temperature-dependent PL spectra from 14 to 300 K, and (**d**) scheme of relaxation processes in self-aligned type-II junction GaSb QD with GaAs capping layer.

To investigate the band alignment between the GaSb QD and GaAs layers, we performed PL measurements. Figure 4a presents the PL spectra of a QD sample measured at 16 K with laser power of 1–30 mW. Considering the peak separation using Gaussian fitting, the PL peak at 1.05 eV, which had a full width at half maximum (FWHM) of approximately 258 meV, was attributed to the GaSb QDs. The energy of the PL peak emission was somewhat lower than those reported for GaSb QDs in previous papers^16, 27–29^. Three peaks were observed at 1.47 eV, 1.24 eV, and 1.32 eV; the first peak was attributed to bulk GaAs, and the other two peaks were attributed to a GaAs_*x*_Sb_1−*x*_ WLL. As the laser power density was increased, the peak at 1.05 eV from the GaSb QDs was observed to blue-shift to 1.13 eV. However, the blue shifts of the other two peaks at 1.24 and 1.32 eV from the GaAs_*x*_Sb_1−*x*_ WLL were weakened, as shown in Fig. 4b. The clear blue-shift behavior with increasing laser power at 1.05 eV was caused by the QD structure; it originated from coulombic interactions and the QD state-filling of holes from the spatial separation of holes in the GaSb QD from the attracted electrons confined in the nearby GaAs regions^16, 30, 31^. However, the peak shifts at 1.24 eV and 1.32 eV showed differences from the nature of QDs.

**Figure 5:**
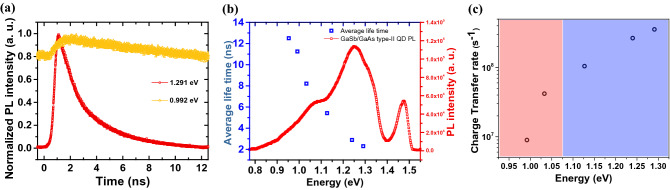
(**a**) TRPL spectra at 300 K and (**b**) average lifetime fitting data based on TRPL. (**c**) Many-state Marcus model fitting data of GaSb QD from 0.75 to 1.55 eV.

To confirm the GaSb QD properties, we investigated the temperature-dependent PL, as shown in Fig. 4c. Some properties were related to the QD structure of the GaSb embedded in the WLL, as shown in Fig. 4c,d. As the temperature increased, an interlayer exciton was generated by the activated phonon owing to the interaction of holes in the GaSb QDs with electrons in the GaAs_*x*_Sb_1−*x*_ WLL. This process was reflected in the energy shift of the interlayer exciton to a lower energy, as shown in Fig. 4c,d. Because the calculated exciton Bohr radius in previously reported papers of ~ 20 nm was similar to the dimension of the GaSb QD shown in Fig. 2^32^, a weak quantum confinement effect was generated in the QD. This effect contributed to the increase in the peak intensity associated with the GaSb QDs at 300 K. In addition, owing to the contribution of the phonon to the interlayer exciton, the PL intensity of the GaAs substrate and GaSb WLL was reduced. These optical characteristics indicated that a type-II band alignment was well formed in the GaSb QD/GaAs heterostructure.To confirm the type-II band alignment of the GaSb QD/GaAs heterostructure, we investigated the carrier transfer mechanism through TRPL measurements at 300 K. Figure 5a presents the decay profile of the PL at 1.29 and 0.99 eV. The decay curve at 0.99 eV indicated mono-exponential behavior, ascribed to the typical type-II staggered band alignment of GaSb QDs; the long decay time was attributed to the reduced spatial overlap between the electrons in the WLL and the holes in the QDs. In comparison with this long decay time, the decay curve of 1.29 eV presented a two-step process comprising a faster initial and slower tail component, as shown in the fitting parameters in Table 1. The average decay times < *t* > of the QDs were estimated as a linear sum of weighted multiple exponentials, where A_i = 1,2,…_ refers to the weighting coefficient for each exponential, and *τ*_i = 1,2,…_ indicates the corresponding fitted decay characteristic times^32^. In our fittings, we used up to I = 2, which provides a reasonable fit for the measured values. The average decay time varied from 2.3 to 12.5 ns from the decay curves at the different peak positions, as shown in Fig. 5b. From the decay times, we can estimate the charge-transfer rate constant *k*_ct_ using the following equation:

1 $$k_{{{\text{ct}}}} \, = \,{1}/t_{{{\text{WLL}}}} \, - \,{1}/t_{{{\text{Tran}}}}$$

where *t*_WLL_ and *t*_Tran_ are the average emission lifetimes of the GaSb WLL and charge transfer, respectively. For the charge-transfer dynamics between a delocalized continuum state and a localized state, for example, between a 2D wetting layer and a 0D QD, the functional form of this many-state Marcus model is as follows:

2 $${k}_{ct}={\int }_{-\infty }^{+\infty }\frac{2\pi }{\hslash }\rho (E){\left|H(E)\right|}^{2}\frac{1}{\sqrt{4\pi \lambda {k}_{B}T}}exp\left(-\frac{{\left(\lambda +\Delta G+E\right)}^{2}}{4\lambda {k}_{B}T}\right)dE$$

where *ρ*(*E*), *H*(*E*), and Δ*G* indicate the density of the accepting state, the electronic coupling, and the energy difference between the donor and acceptor energy levels, respectively^33–35^. The density of states *ρ*(*E*) obviously differs between a 3D WLL (3D) and a 0D QD. The electronic coupling, *H*(*E*), which depends on the physical overlap between the transferred electron in its initial and final states, can be independent of the energy because of the physical structure. Δ*G* can be expressed as Δ*G* = *E*_*h*(QD)_ − *E*_*h*(WLL)_, where *E*_*h*(QD)_ and *E*_*h*(WLL)_ are the energies of the holes in the QD and WLL, respectively. Because the PL spectrum is strongly related to the hole energies of the WLL and QD in a type-II system, Δ*G* is assumed to be linearly related to energy. As shown in Fig. 5c, the charge-transfer rate linearly increased in the overall range, whereas the slope in the QD region changed drastically. This sudden change originated from the transition of *ρ*(*E*) between the WLL and the QD. As a result, we verified that the GaSb QDs were well grown on the GaAs substrate and that the GaSb QDs had a type-II band structure with GaAs.

**Table 1:** Fitting parameters of TRPL data at each energy.

Energy (eV)	A_1_	τ_1_ (ns)	A_2_	τ_2_ (ns)	Average life time (ns)	Charge transfer rate
1.291	29,114	0.468	10,386.2	3.081	2.300663	3.54721E+08
1.24	28,037	0.863	9521.1	4.142	2.895174	2.65466E+08
1.127	7134	1.264	11,265.1	5.979	5.422287	1.04488E+08
1.033	887	1.687	4745	8.454	8.21065	4.18570E+07
0.992	0	0	2556.9	11.248	11.248	8.96864E+06
0.953	0	0	1037.2	12.510	12.51 (t_QD_)	0

**Figure 6:**
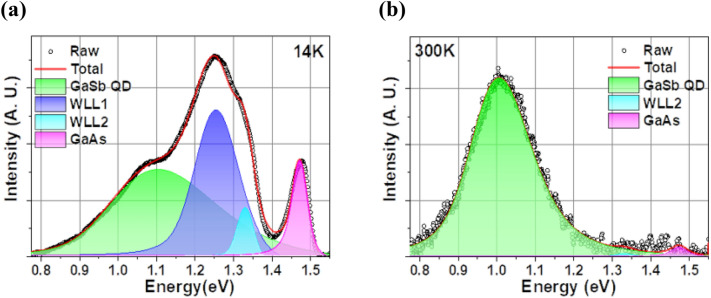
Fitting data of linear-scale PL spectra at (**a**) 14 K and (**b**) 300 K for calculating E-IQE based on comparing two areas of GaSb QD.

Following a previous study^36^, the PL data at 14 K were fitted with four peaks to calculate the E-IQE of the GaSb QDs, as shown in Fig. 6a,b; the peaks corresponded to the GaSb QD, WLL1, WLL2, and GaAs. However, the PL data at 300 K, fitted with one or two peaks, indicated that the non-radiative emission process was increased at high energies, thus indicating effective charge transfer to the GaSb QDs. Finally, we extracted the E-IQI of 15 ± 0.2% using the ratio of the GaSb QD fitting area. In conclusion, the self-grown GaSb QDs on GaAs with a well-defined structure had a high E-IQE. To confirm the relationship between E-IQE and lattice strain, we calculated the total band structure depending on the lattice strain in GaSb, as shown in Figure S4a,b. Based on the simulation data, the band gap of GaSb with a 1.8% strain along the [111] direction was smaller than that of GaSb without strain. Specifically, the valance-band degeneracy is broken by the lattice strain, and the band gap with strain becomes smaller than that without strain, as shown in Figure S4c,d, which is consistent with the red-shift of the QD PL. In general, the reported results indicate that the maximum efficiency of a QD is decreased within the Shockley–Queisser limit, that is, the lattice strain reduces the efficiency^37^. However, because the lattice strain from the self-grown GaSb QDs in this study was minimized, the efficiency was optimized by controlling the lattice strain. In this self-grown QD formation, where the interfacial strain was minimized by changing the growth direction, the self-aligned type-II band structure with an optimized strain affected the QD size and the formation of an effective band structure, resulting in an improved efficiency compared to those of samples with higher strain reported in other papers^38, 39^.

## Conclusion

In summary, we investigated the growth mechanism of type-II GaSb QDs grown on GaAs using a self-grown nanowire process. During the growth, a Ga droplet was formed on top of the GaAs layer, and a hexagonal GaSb QD with a zinc-blende structure grew through a nanowire growth mechanism by the diffusion of Sb into the Ga droplet. Based on the PL data, we confirmed the state-filling effect in GaSb QDs with type-II band alignment. In addition, using TRPL fitting data, we obtained two types of decay time constants related to nonradiative and radiative decay, which were consistent with the band alignment type. Moreover, the formation of GaSb QDs with type-II band alignment with a GaAs layer through the self-growth nanowire-like mechanism decreased the interfacial strain through changing the growth direction, resulting in an increase in the E-IQE up to 15%. This study suggests the possibility of self-grown GaSb QDs following the nanowire growth mechanism, which can successfully improve the optical characteristics of GaSb QDs. The GaSb QDs can be self-grown using a GaSb droplet on GaAs, considering the band alignment. Furthermore, the synthesis method is useful for optimizing the efficiency of various optical devices utilizing QDs. For example, the GaSb QDs grown in this study can be used as near-infrared sensors by bandgap tuning using the lattice strain.

## Methods

GaSb QDs were grown on n-type GaAs(001) substrates in a RIBER compact-21E MBE system equipped with cryogenic and ion getter pumps. The system was also equipped with a RIBER VAC500 valved cracker cell and VEECO Sb valved cracker cell to supply the As tetramer and Sb dimer elements, respectively. Ga, the group-III metal source, was supplied through an effusion cell. The flux of the group-III material was fixed at a specific value during the growth process. The top-view images of the QD samples were obtained using SEM. To analyze the nanostructure, morphology, and thickness of the GaSb QD, GaAs buffer layer, and GaAs SPS layer, we used an FEI Tecnai F-20 h-TEM at an accelerating voltage of 300 kV. The SAD patterns were processed using FFT to analyze the well-formed crystal structure in the GaSb QD/GaAs heterostructure. To confirm the type-II band alignment of the GaSb QD/GaAs heterostructure, PL measurements were performed using a 532-nm diode-pumped solid-state laser (30 mW, CNI laser) and a monochromator (SP-2300, Princeton Instruments) with a closed-cycle refrigerator (RW-3, Leybold). To investigate the carrier transfer mechanism, we measured the TRPL at 300 K using a time-correlating single-photon counting module (Picoquant, PH-300) with an 800-nm Ti:sapphire laser (80 MHz, Mai-tai, Spectra-physics) and a near-infrared photomultiplier tube (Hamamatsu, H10330c-75). To predict the effect of strain on the GaSb QDs, density functional theory (DFT) calculations were performed using the Vienna ab-initio simulation package (VASP). A 500-eV cut-off energy and 9 × 9 × 9 k-point grid were used for the DFT calculations to achieve a *k*-point spacing of less than 0.2 (1/Å) with the Perdew–Burke–Ernzerhof functional revised for solids (PBEsol). The GaSb unit cell was geometrically optimized until convergence was achieved at 0.005 eV/Å. To simulate the strain effect on the GaSb QD, the tensile strain of 1.8% in the [111] direction of the GaSb unit cell ([001] direction of the re-defined hexagonal cell) was applied, based on the measured strain in the TEM images. The lattice parameter in the other directions ([100] and [010] of the re-defined hexagonal-like cell) was relaxed until the stress in those directions was smaller than 0.01 GPa. During relaxation, a 500-eV cut-off energy and 9 × 9 × 3 k-point grid were used. To obtain an accurate band structure for the strained GaSb, we performed DFT calculations with the hybrid functional Heyd–Scuseria–Ernzerhof 06 (HSE 06). A 500-eV cut-off energy and 9 × 9 × 9 k-point grid were used to calculate the GaSb band structure.

## Supplementary Information

Supplementary Information.
