# Supplementary material for: Optical characteristics of type-II hexagonal-shaped GaSb quantum dots on GaAs synthesized using nanowire self-growth mechanism from Ga metal droplet
Source: Sci Rep. 2021 Apr 8;11:7699. doi: 10.1038/s41598-021-87321-9 (PMC8032789; doi:10.1038/s41598-021-87321-9)
Supplement: Supplementary file 1 — Supplementary Information. [file 41598_2021_87321_MOESM1_ESM.docx]

Optical characteristics of type-II hexagonal shaped GaSb Quantum dots on GaAs synthesized using nanowire self-growth mechanism from Ga metal droplet

Min Baik^1,2^, Ji-hoon Kyhm^3^, Hang-Kyu Kang^1,2^, Kwang-Sik Jeong^1^, Jong Su Kim^4^, Mann–Ho Cho^1,^*, Jin Dong Song^2,^*

^1^ Department of Physics, Yonsei University, Seoul 03722, Korea

^2^ Center of Opto-Electronic Convergence Systems, Korea Institute of Science and Technology, Seoul 02792, Korea

^3^ Quantum functional semiconductor research center, Dongguk University, Seoul 04620, Korea

^4^ Department of Physics, Yeungnam University, Gyeongsan 38541, Korea

Keywords: GaSb; Type-II band alignment; Self-growth; Photoluminescence; Droplet epitaxy; Nanowire

**Supporting information**


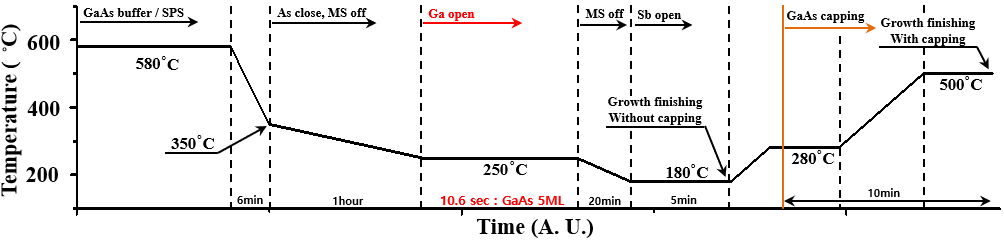


Figure S1. Growth process of GaSb QD on GaAs by Droplet epitaxy using Molecular Beam Epitaxy system


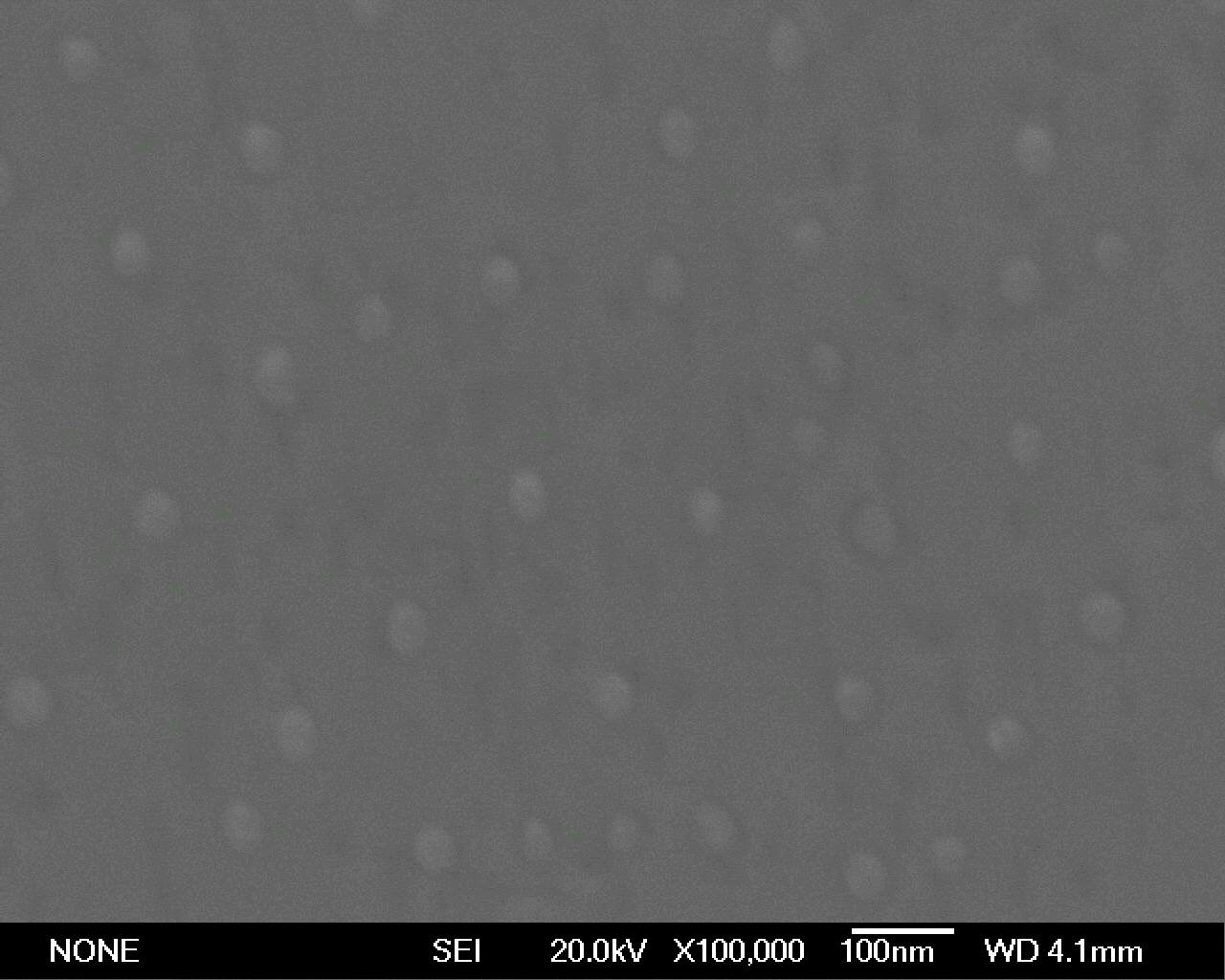


Figure S2. SEM top-view image of Ga droplets


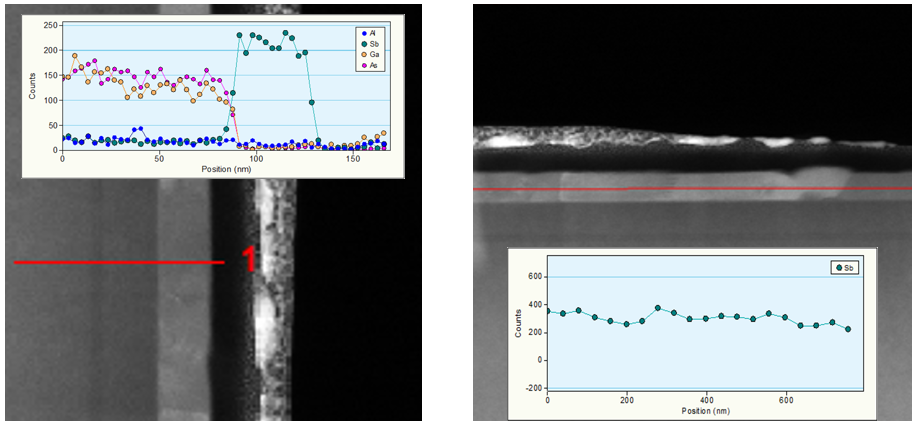


Figure S3. Cross-section images and EDX data of Sb layer on GaAs outside area of GaSb QDs shown in figure 2


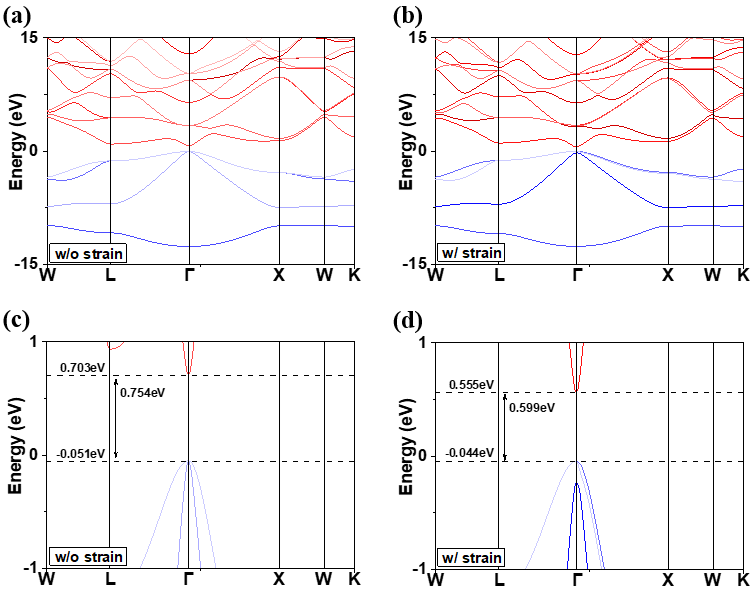


Figure S4. DFT calculation data of the GaSb band structure (a) without strain and (b) with a 1.8% strain along the [111] direction, and band gap of GaSb (c) without strain and (d) with a 1.8% strain along the [111] direction; the band gap is narrowed by strain along the [111] direction
